# Migraine and major depression: localizing shared genetic susceptibility in different cell types of the nervous systems

**DOI:** 10.3389/fneur.2023.1254290

**Published:** 2023-11-15

**Authors:** X. Michelle Androulakis, Xuanxuan Yu, Xia Zhu, Melinda A. Thiam, Guoshuai Cai

**Affiliations:** ^1^Department of Neurology, Columbia VA Healthcare System, Columbia, SC, United States; ^2^Department of Epidemiology and Biostatistics, Arnold School of Public Health, University of South Carolina, Columbia, SC, United States; ^3^Department of Environmental Health Science, Arnold School of Public Health, University of South Carolina, Columbia, SC, United States; ^4^Department of Psychiatry, New Mexico VA Healthcare Care System, Albuquerque, NM, United States; ^5^Department of Surgery, College of Medicine, University of Florida, Gainesville, FL, United States

**Keywords:** migraine, depression, CNS, PNS, cell-type, expression

## Abstract

**Background:**

There is a bidirectional relationship between migraine and major depression disorder (MDD). They likely share important risk genes associated with different cell types in the central nervous system (CNS) and peripheral nervous system (PNS). Profiling the expression of these genes in specific cell types is critical in understanding the pathophysiology of the relationship between migraine and MDD.

**Methods:**

Associated genes shared by migraine and MDD were identified by consolidating multiple curations of human disease-gene associations. Subsequently, the expression of overlapping genes was profiled and compared across the different cell types in CNS, PNS and neurovascular cells using eight single cell RNA sequencing datasets, including two human CNS datasets, two mouse CNS datasets, one human PNS dataset and three mouse PNS datasets.

**Results:**

45 shared genes between migraine and MDD were identified. Consistently found in all eight datasets, dopaminergic and serotonergic neurotransmitters were broadly expressed in CNS and PNS cell types. Glutamatergic and endocannabinoid genes were specifically expressed in CNS neurons and astrocytes. Synthesis and/or Release and Binding of Neuropeptides were specifically expressed in PNS peptidergic nociceptor (PEP). Genes related to inflammatory factors and immune responses were specifically expressed in CNS microglia. Among which, *IL1B* and *COMT* were highly expressed in CNS microglia cells.

**Conclusion:**

Single cell RNA sequencing of the CNS and PNS helps to identify the shared genes between migraine and MDD that are enriched in specific cell types. The findings provide new insight in understanding the underlying mechanism of action for the bidirectional co-morbidity between migraine and MDD.

## Background

Both migraine and major depression disorder (MDD) are among the most prevalent and debilitating diseases worldwide. Migraine and MDD frequently coexist and share a bidirectional relationship (1–3): migraine predicts the first onset of MDD, and MDD increases risk of the first onset of migraine (1). Interestingly, this relationship is not observed in non-migraine severe headache types (4). In patients with MDD, risk of co-morbid migraine is 2–3 times higher and individuals with migraine have more than threefold risk of depression (5). Besides, concurrent migraine and MDD is associated with higher disability and more resistant to conventional pharmacotherapy (6), resulting in decreased quality of life, poorer response to treatment, overall worse prognosis, and high socioeconomic burden for patients, their families, and society.

Both migraine and MDD overlap in clinical manifestation, health outcome and genetic risk factors. The exact mechanism underlying the shared pathogenesis of comorbidity between migraine and MDD is unknown, but likely involving dysfunctional CNS and PNS. There is a paucity of data on how the cell types regulated by specific genes will lead to migraine and depression. Indeed, migraine and MDD are associated with deficiency in regulating excitatory-inhibitory balance in CNS (7, 8). Astrocytes with unusual low level of ɑ2 Na(+), K(+) ATPase were thought to be a risk factor for familial hemiplegic migraine type 2 (9). Different expression levels of astrocyte– and oligodendrocyte-related genes between migraine with and without aura have been identified (10). Activation of microglia and astrocytes produces and releases neuroexcitatory substances which may lead to neural hypersensitivity, and blocking these glial cell activation is capable of relieving migraine (11). Significant reductions in astrocytes density and expression of their markers without associated neuronal loss is unique in MDD but not commonly observed in other neuropsychiatric and neurodegenerative disorders (12). Other studies showed that changes of oligodendrocyte and oligodendrocyte precursor cells (OPC) were associated with MDD (13). Lastly, neurovascular unit dysfunction was found to contribute to migraine and MDD in two separate studies (14, 15). Therefore, Profiling the expression of disease associated genes in specific cell types is critical in better understanding pathophysiology underlying these two comorbid conditions and developing new therapeutic targets (16).

In this study, we propose to identify and localize the expression of shared associated genes in specific cell types in CNS and PNS between migraine and depression. Eight CNS/PNS single-cell RNA sequencing datasets were analyzed, including two human CNS datasets, two mouse CNS datasets, one human PNS dataset, and three mouse PNS datasets. We aim to provide genetic evidence with regards to cell type-specific roles of the identified shared susceptibility genes between migraine and depression. The data that are produced in this study provide new insights into the cell-type specific drivers underlying the mechanisms of migraine and MDD and the links between them. It provides important directions for further functional analysis including *in-vivo* studies using mouse models, such as the cortical spreading depression (CSD) (17) and the inflammatory soup (IS) (18) headache models.

## Methods

### Identification of associated genes

MalaCards database (19) was used to search for genes which are associated with migraine and MDD, separately through two disease cards of “Migraine With or Without Aura (MA)” and “Major Depressive Disorder (MDD).”

### Datasets

Four CNS and four PNS datasets were analyzed in this study, including two human CNS datasets (20, 21) (GSE67835, 267 cells; GSE97930, 33,722 cells), two mouse CNS datasets (22, 23) (the Mouse Brain Atlas, 95,753 cells; GSE60361, 1,667 cells), three mouse PNS datasets (22, 24, 25) (the Mouse Brain Atlas, 2,311 cells; GSE101984, 3,688 cells; GSE197289, 67,991), and a human PNS dataset (25) (GSE197289, 17,223 cells). The mouse PNS dataset, GSE197289, are from mouse of normal models (PBS) and mouse of two headache models (CSD, IS), while all other datasets are from normal tissue. In CNS and PNS datasets, we studied cell types that considered to be involved in migraine pathophysiology, including CNS cell types (neurons, oligodendrocytes, microglia, astrocytes); PNS cell types (peptidergic nociceptors (PEP), non-peptidergic nociceptors (NP), large diameter neurofilament-positive mechanoreceptors (NF), C low threshold mechanoreceptors (cLTMR), Satellite cells, Schwann cells); and neurovascular endothelial cells (vasc/endoth).

**GSE60361**: Zeisel et al. (22) characterized cells from both wild type and transgenic mice between postnatal 21 and 31 days to study the cell types in the mouse cortex and hippocampus. Cells in somatosensory cortex or CA1 hippocampal brain regions were processed by Fluidigm C1 and sequenced by using Illumina HiSeq 2000 platform. A total of 3,315 cells that met the criteria of passing visual inspection of the images and containing more than 2,500 RNA molecules were subjected to clustering using the BackSPIN method, resulting in 9 major cell types identified in the original study. In this study, 1,667 cells of four CNS cell types and vasc/endoth cells were focused as shown above.

**GSE67835**: To investigate the cellular diversity in the human brain, Darmanis et al. (20) sequenced 482 cells in cortical tissue from eight healthy adults and four embryonic samples at the whole transcriptome level using Fluidigm C1 and Illumina NextSeq 500. After filtering out cells with less than 400,000 reads, 466 cells were kept for clustering. Specifically, dimension reduction of the distance between cells was performed, followed by the parametric Gaussian mixture model for cell type identification in the original study. 267 cells from healthy adults of interested CNS cell types were included in our study.

**GSE97930**: To characterize the cell types in human brain, cells in the visual cortex, frontal cortex, and lateral cerebellar hemisphere from six adult human postmortem brain samples were sequenced using snDrop-seq (21) and Illumina HiSeq 2,500. Expression measurements for a total of 35,289 cells were retained in the three brain regions after filtering out cells with read count less than 3,000. Clustering and cell type identification were performed separately for each brain region using PAGODA2 R package. We included10,319 cells of five cell types in the front cortex in our analysis.

**GSE101984**: Nguyen (24) sequenced cells from the trigeminal ganglion in healthy mice using the Drop-seq technique and Illumina MiSeq. Cells with a gene count of fewer than 200 or greater than 8,500, as well as those exhibiting mitochondrial transcripts exceeding 0.3%, were excluded, leaving 6,998 cells. The Seurat package was used to cluster the cells, and non-somatosensory cells were removed. This process resulted in 3,943 neurons being further clustered into the primary PNS cell types in the original study. We analyzed six cell types comprising 3,688 cells in this study.

**GSE197289**: Yang et al. (25) conducted a snRNA-seq study including 14 mice trigeminal ganglion biological replicates from one normal model (PBS) and two headache models (CSD, IS), and 3 human donors without neurological diseases. Specifically, 59,921 mouse cells and 38,028 human cells were sequenced using 10X Genomics V3.1 Gene Expression Assay and NextSeq 550 platform. Cells with more than 400 unique genes, less than 15,000 total UMIs, and less than 5% of the mitochondrial counts were included for subsequent clustering conducted by using the Seurat package. Filtering out PNS cell types out of the study focus, we included in the analysis 17,223 human cells and 67,991 mouse cells.

**Mouse brain atlas**: To create an extensive census of cell types within the mouse nervous system, Zeisel et al. (22) performed a comprehensive study to characterize the single-cell transcriptomes of the adolescent mouse nervous system. Cells were sequenced by 10x Genomics Chromium Single Cell Kit v.1. Cells with less than 600 UMIs, or less than 1.2-fold molecule to gene ratio were excluded. In addition, Genes found in less than 20 cells or present in over 60% of all cells were eliminated. The cytograph package was used to cluster cells into CNS, PNS, and non-neuronal cells. 2,311 PNS cells and 95,753 CNS cells were focused in our study.

### Data processing and cell type identification

The sequencing counts of single cell RNA-seq datasets were processed using Seurat (26). Cell types in the mouse Brain Atlas datasets, the mouse CNS dataset (GSE60361), the human CNS datasets (GSE67835, GSE97930), the PNS datasets of human and mouse cells (GSE197289) were identified by their original studies (20–23, 25), respectively. For all data, log cpm (counts per million) were calculated by “NormalizeData” function and then centered and scaled by its standard deviation. For the mouse dataset of trigeminal ganglion neurons (24) (GSE101984), the principal component analysis (PCA) was performed on the top 2000 variable genes identified by “FindVariableFeatures” function. Cells were assigned into 13 clusters using “FindClusters” function with the top 20 PCs as the inputs and resolution was set as 0.3. The average expressions and expressed percentages of marker genes used by the Mouse Brain Atlas study (22) were used to identify NF, satellite, endothelial, cLTMR, schwann, PEP, NP cell types.

### Differential expression analysis

The Wilcox rank sum test implemented in “Seurat” was performed to compare gene expression in a certain cell type with that in all other cell types, or to compare the gene expression between healthy and headache mouse models in each cell type. Further, Bonferroni method was applied to adjust 𝑝 values to control false positives due to multiple comparison. A significant difference was detected when three Seurat default criteria were satisfied, including adjusted 𝑝 value was less than 0.05, log fold change was larger than 0.25, and expression was detected in more than 10% of cells.

## Results

### Common genetic risk factors between Migraine and MDD

169 migraine-related and 125 depression-related genes were identified from MalaCards database (see Methods). 45 of them were overlapped and categorized into 8 functional groups: synthesis and/or release of binding of neuropeptides (*n* = 7), dopaminergic and serotonergic neurotransmitters (*n* = 17), glutamatergic neurotransmitters and endocannabinoid genes (*n* = 5), calcium channel (*n* = 1), inflammatory factors and immune responses (*n* = 4), hormone related genes (*n* = 6), vascular function regulation (*n* = 3), and drug metabolism (*n* = 2). The genes in each category were shown in Table 1.

**Table 1:** Biological group and effect of 45 overlapped genes related to migraine and depression.

Group	Gene Symbol	Description
Synthesis and/or Release of Binding of Neuropeptides	*HCRT*	Hypocretin Neuropeptide Precursor
	*NGF*	Nerve Growth Factor
	*NPY*	Neuropeptide Y
	*BDNF*	Brain Derived Neurotrophic Factor
	*ADCYAP1*	Adenylate Cyclase Activating Polypeptide 1
	*TAC1*	Tachykinin Precursor 1
	*TACR1*	Tachykinin Receptor 1
Dopaminergic and Serotonergic Neurotransmitters	*COMT*	Catechol-O-Methyltransferase
	*DRD1*	Dopamine Receptor D1
	*DRD2*	Dopamine Receptor D2
	*DRD3*	Dopamine Receptor D3
	*DRD4*	Dopamine Receptor D4
	*MAOA*	Monoamine Oxidase A
	*MAOB*	Monoamine Oxidase B
	*SLC6A3*	Solute Carrier Family 6 Member 3
	*SLC6A4*	Solute Carrier Family 6 Member 4
	*HTR1A*	5-Hydroxytryptamine Receptor 1A
	*HTR1B*	5-Hydroxytryptamine Receptor 1B
	*HTR1D*	5-Hydroxytryptamine Receptor 1D
	*HTR2A*	5-Hydroxytryptamine Receptor 2A
	*HTR2B*	5-Hydroxytryptamine Receptor 2B
	*HTR2C*	5-Hydroxytryptamine Receptor 2C
	*HTR3A*	5-Hydroxytryptamine Receptor 3A
	*TPH1*	Tryptophan Hydroxylase 1
Glutamatergic Neurotransmitters and Endocannabinoid Genes	*GRIN2B*	Glutamate Ionotropic Receptor NMDA Type Subunit 2B
	*GRM5*	Glutamate Metabotropic Receptor 5
	*SLC1A2*	Solute Carrier Family 1 Member 2
	*SLC1A3*	Solute Carrier Family 1 Member 3
	*CNR1*	Cannabinoid Receptor 1
Calcium Channel	*CACNA1C*	Calcium Voltage-Gated Channel Subunit Alpha1 C
Inflammatory Factors and Immune Responses	*CRP*	C-Reactive Protein
	*IL1B*	Interleukin 1 Beta
	*IL6*	Interleukin 6
	*TNF*	Tumor Necrosis Factor
Hormone Related Genes	*CRH*	Corticotropin Releasing Hormone
	*OXT*	Oxytocin/Neurophysin I Prepropeptide
	*POMC*	Proopiomelanocortin
	*PRL*	Prolactin
	*LEP*	Leptin
	*OPN4*	Melanopsin
Vascular regulators	*ACE*	Angiotensin I Converting Enzyme
	*MTHFR*	Methylenetetrahydrofolate Reductase
	*NOS1*	Nitric Oxide Synthase 1
Drug metabolism	*OPRM1*	Opioid Receptor Mu 1
	*CYP2D6*	Cytochrome P450 Family 2 Subfamily D Member 6

### Expression profiles of risk genes in CNS cells

**Figure 1:**
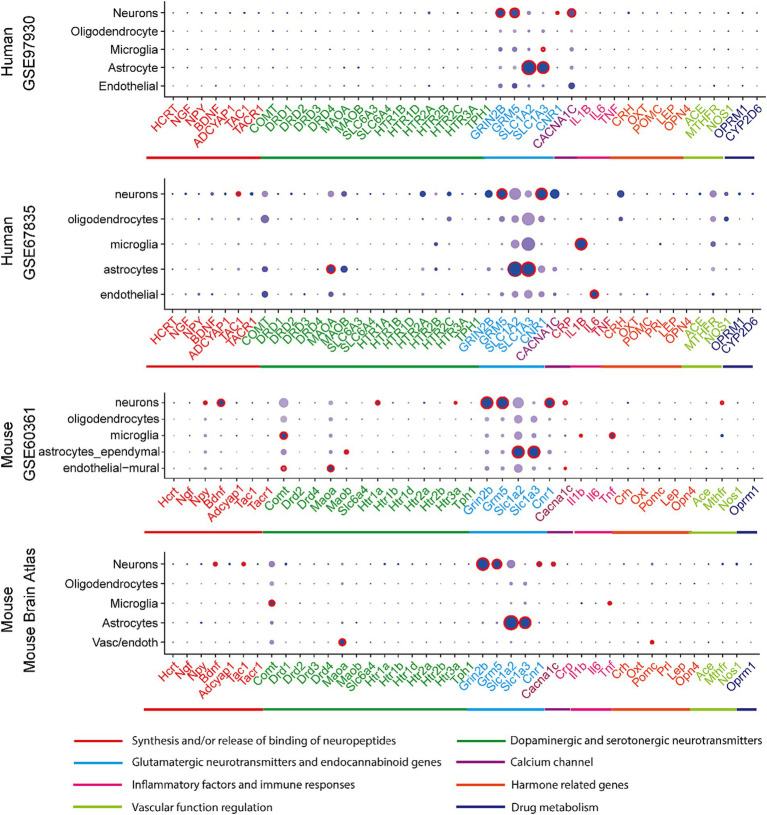
Expression profiles in CNS cell types in human and mouse datasets. In a particular cell type, the expression of genes was illustrated in 8 categories indexed by color bars. A larger dot size indicates a higher detection rate of gene expression within each cell type, while a darker color indicates a higher average level of expression. The red circles indicate significantly higher expression in a cell type compared to all others.

The expression of above 45 associated genes in individual cells were profiled in both human and mouse CNS (Figure 1). In the human and mouse CNS, genes including those in activities of neuropeptides and neurotransmitters (Synthesis and/or Release and Binding of Neuropeptides, Dopaminergic and Serotonergic Neurotransmitters, Glutamatergic neurotransmitters and endocannabinoid genes) were broadly expressed in neurons (Figure 1; Supplementary Table S1), which reflect the strong association of neurons and these two neurological problems.

In at least one human dataset and one mouse dataset, cross-species high expression was observed on genes related to NMDA receptor (GRIN2B), metabotropic glutamate receptor (GRM5), cannabinoid receptor (CNR1), substance P and neurokinin A (TAC1), the flow of calcium ions (CACN1C) in neurons; and glutamate transporting process (SLC1A2 and SLC1A3) in astrocytes; as well as inflammatory response (IL1B) in microglia. Notably, the expression of glutamate transporter genes (SLC1A2 and SLC1A3) in astrocyte and glutamate receptor 5 gene (GRM5) in neurons are consistently higher expressed across all four datasets (Supplementary Table S3).

### Expression profiles in PNS cells

**Figure 2:**
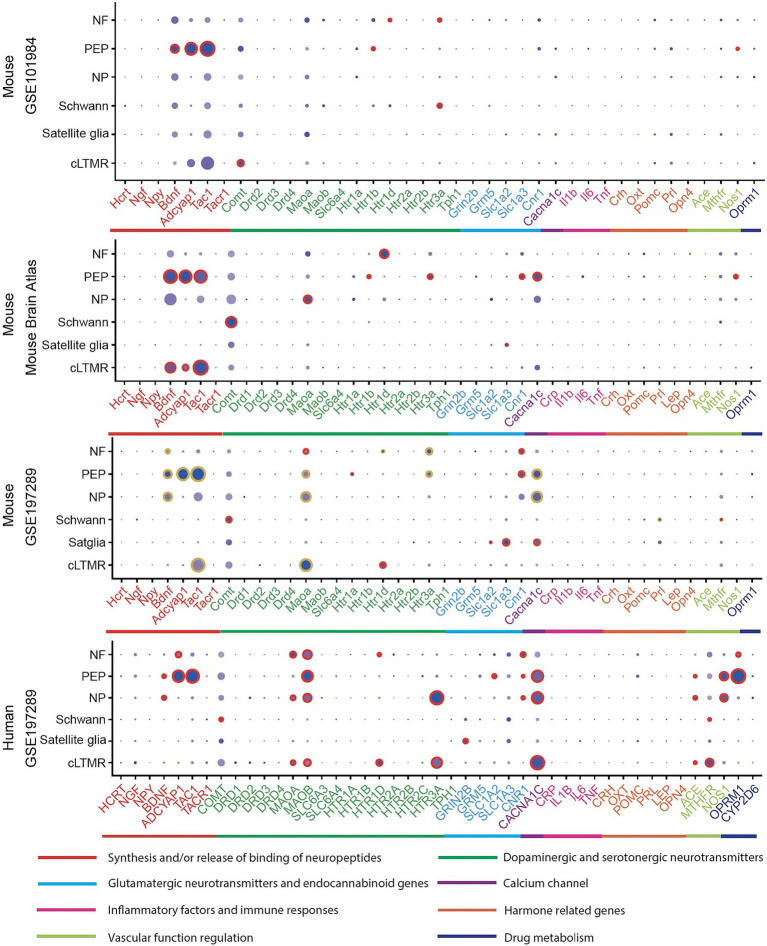
Expression profiles in PNS cell types in human and mouse datasets. In a particular cell type, the expression of genes was illustrated in 8 categories indexed by color bars. A larger dot size indicates a higher detection rate of gene expression within each cell type, while a darker color indicates a higher average level of expression. The red circles indicate significantly higher expression in a cell type compared to all others. The golden circles in the GSE197289 profile indicate significantly differential expression between normal and headache models.

The expression of the 45 associated genes in individual cells of normal PNS were profiled in three mouse and one human datasets (Figure 2; Supplementary Table S2).

In at least one human dataset and one mouse dataset, cross-species high expressions were observed on genes related to MAOA, serotonergic neurotransmitters (HTR1D), cannabinoid receptors (CNR1) in NF, neuropeptides activity (BDNF, ADCYAP1, TAC1), CACNA1C, NOS1 in PEP, BDNF and CACNA1C in NP, COMT and MTHFR in Schwann cells, HTR1D in cLTMR. Notably, most of these signatures were differentially expressed in their corresponding cell types of mouse normal and headache models (Figure 2, GSE197289), which highlighted their cell-type specific roles in migraine. Across all human and mouse datasets, significantly high expressions were observed for *HTR1D* in NF, *BDNF, ADCYAP1, and TAC1* in PEP (Supplementary Table S3).

## Discussion

Genes associated with migraine and depression are identified and their expressions were studied in different cell types in CNS, PNS, and neurovascular cells. Results are consistent with previous studies, that some associated genes are significantly enriched within specific cell types, indicating that the dysregulation of these genes may lead to corresponding dysfunctional cell types that contribute to migraine or depression (27). Some genes are expressed in multiple cell types, e.g., *MAOA* was significantly highly expressed in astrocytes and microglia of CNS and large diameter neurofilament-positive mechanoreceptors of PNS, while *CACNA1C* was significantly highly expressed in neurons and neurovascular cells of CNS and non-peptidergic nociceptors of PNS. Thus, individuals possessing these genes may have multiple dysfunctional cell types contributing to migraine or depression. Indeed, these cell-type specific genes were found differentially expressed in the PNS of normal and headache models. Polygenic analysis in CNS and PNS cell types may reveal pathophysiology evidence and help stratify patients through routine genotyping (28).

The findings of this study underscore the important roles of glutamatergic system in neurons and astrocytes, genes related to synthesis and/or release and binding of neuropeptides in PEP and cLTMR implicated in both migraine and MDD. Additionally, in CNS cell types, the dysregulation of endocannabinoid system, substance P, and calcium iron flow in neurons, as well as IL 1 in microglia may attribute to the association between migraine and MDD. Similarly, in PNS cell types, the dysregulation of dopaminergic system, calcium iron flow in NF, PEP, NP and cLTMR may also be related with migraine and MDD. We propose that these genes in specific CNS and PNS cell types have potential to serve as biomarkers for monitoring treatment response in patients with comorbid migraine and MDD. On the other hand, clinicians may consider available migraine drugs targeting glutamatergic pathways such as ketamine, topiramate, and memantine for refractory migraine with MDD. High cost and side effect profile of repeated ketamine infusion therapy warrant using genetic/molecular biomarkers to predict treatment response. In addition, medication mediating flow of calcium ion across blood brain barrier may have potential to benefit both migraine and MDD. Lastly, the consistent pattern across mouse and human data implies the feasibility of using animal models for further mechanism study and developing novel therapy targeting associated CNS or PNS cell types.

Although current analyses are performed on eight single-cell RNA-seq datasets, limitations exist when we interpret the results. First, the associated genes were identified only based on genomic data, more omics data may be included to validate the results. Moreover, although genome-wide association studies utilized scRNA-seq data from mice to approximate the gene expression profiles of brain cell types in humans (29), more human CNS and PNS datasets are needed in the future upon the availability of a large scRNA-seq dataset. Based on such a dataset, we can validate our findings by comparing the gene expressions between healthy humans and patients with MDD or migraine, with the adjustment of the confounding effects from factors such as age and sex.

## Data availability statement

The datasets presented in this study can be found in online repositories. The names of the repository/repositories and accession number(s) can be found in the article/Supplementary material.

## Ethics statement

Ethical approval was not required for the study involving humans in accordance with the local legislation and institutional requirements. Written informed consent to participate in this study was not required from the participants or the participants' legal guardians/next of kin in accordance with the national legislation and the institutional requirements.

## Author contributions

XA: Conceptualization, Funding acquisition, Investigation, Resources, Supervision, Writing – original draft, Writing – review & editing. XY: Data curation, Investigation, Formal analysis, Visualization, Writing – review & editing. XZ: Writing – review & editing, Data curation. MT: Investigation, Writing – review & editing. GC: Coceptualization, Funding acquisition, Investigation, Resources, Methodology, Formal analysis, Visualization, Supervision, Writing – original draft, Writing – review & editing.
