# Supplementary material for: Migraine and major depression: localizing shared genetic susceptibility in different cell types of the nervous systems
Source: Front Neurol. 2023 Nov 15;14:1254290. doi: 10.3389/fneur.2023.1254290 (PMC10684773; doi:10.3389/fneur.2023.1254290)
Supplement: Supplementary file 1 [file Data_Sheet_1.docx]

**Table S1.** Significant differentailly expressed genes comparing to other cell types in CNS datasets

| Data | Cluster | Gene | Log2  Fold change | P-value | Average  Expression | Expression  Percentage (%) |
| --- | --- | --- | --- | --- | --- | --- |
| GSE97930 | Astrocyte | *SLC1A2* | 4.99 | 0.00ⅹ10^0^ | 6710.00 | 88.47 |
|  |  | *SLC1A3* | 4.58 | 0.00ⅹ10^0^ | 2640.00 | 72.46 |
|  | Microglia | *SLC1A3* | 0.45 | 1.60ⅹ10^-1^ | 405.00 | 18.72 |
|  | Neurons | *GRM5* | 1.60 | 4.84ⅹ10^-246^ | 900.00 | 58.30 |
|  |  | *GRIN2B* | 1.60 | 1.55ⅹ10^-224^ | 716.00 | 52.69 |
|  |  | *CACNA1C* | 1.39 | 1.77ⅹ10^182^ | 620.00 | 48.37 |
|  |  | *CNR1* | 2.88 | 2.52ⅹ10^-62^ | 162.00 | 12.54 |
| GSE67835 | Endothelial | *IL6* | 1.39 | 9.33ⅹ10^24^ | 1.64 | 55.00 |
|  | Astrocytes | *SLC1A2* | 4.91 | 2.51ⅹ10^-31^ | 49.40 | 100.00 |
|  |  | *SLC1A3* | 3.29 | 1.52ⅹ10^-30^ | 16.40 | 100.00 |
|  |  | *MAOA* | 0.61 | 2.18ⅹ10-^5^ | 0.68 | 56.45 |
|  | Microglia | *IL1B* | 2.44 | 7.55ⅹ10^-26^ | 4.41 | 81.25 |
|  | Neurons | *CNR1* | 1.44 | 1.60ⅹ10^-18^ | 2.02 | 79.39 |
|  |  | *GRM5* | 0.46 | 2.91ⅹ10^-17^ | 0.46 | 69.47 |
|  |  | *TAC1* | 0.39 | 1.96ⅹ10^-2^ | 0.32 | 24.43 |
| GSE60361 | Endothelial | *MAOA* | 2.49 | 3.68ⅹ10^-24^ | 193.00 | 50.64 |
|  |  | *COMT* | 0.30 | 3.45ⅹ10^-4^ | 155.00 | 35.74 |
|  |  | *CACNA1C* | 0.74 | 7.80ⅹ10^-2^ | 35.40 | 10.21 |
|  | Astrocytes | *SLC1A3* | 4.92 | 2.69ⅹ10^-174^ | 5260.00 | 98.21 |
|  |  | *SLC1A2* | 4.04 | 5.50ⅹ10^-114^ | 9410.00 | 98.66 |
|  |  | *MAOB* | 3.53 | 2.00ⅹ10^-33^ | 113.00 | 28.13 |
|  | Microglia | *TNF* | 6.76 | 3.69ⅹ10^-133^ | 475.00 | 41.84 |
|  |  | *IL1B* | 6.05 | 2.89ⅹ10^-25^ | 197.00 | 15.31 |
|  |  | *COMT* | 0.89 | 2.88ⅹ10^-01^ | 233.00 | 57.14 |
|  | Neurons | *GRM5* | 3.48 | 0.00ⅹ10^0^ | 758.00 | 97.48 |
|  |  | *GRIN2B* | 2.54 | 0.00ⅹ10^0^ | 882.00 | 98.53 |
|  |  | *BDNF* | 3.35 | 9.31ⅹ10^-176^ | 114.00 | 56.82 |
|  |  | *CNR1* | 3.11 | 3.92ⅹ10^-175^ | 578.00 | 74.94 |
|  |  | *HTR1A* | 3.56 | 7.95ⅹ10^-98^ | 29.70 | 30.65 |
|  |  | *CACNA1C* | 0.37 | 1.34ⅹ10^-34^ | 24.60 | 28.56 |
|  |  | *MTHFR* | 0.59 | 5.05ⅹ10^-25^ | 14.50 | 20.76 |
|  |  | *HTR3A* | 3.94 | 2.30ⅹ10^-24^ | 493.00 | 13.82 |
|  |  | *NPY* | 3.34 | 8.44ⅹ10^-04^ | 1260.00 | 21.87 |
| Mouse Brain Atlas | Vasc/  endoth | *MAOA* | 1.32 | 0.00ⅹ10^0^ | 2.23 | 41.66 |
|  |  | *POMC* | 0.53 | 3.38ⅹ10^-153^ | 0.67 | 15.27 |
|  | Astrocytes | *SLC1A2* | 4.18 | 0.00ⅹ10^0^ | 59.60 | 95.14 |
|  |  | *SLC1A3* | 4.09 | 0.00ⅹ10^0^ | 21.80 | 76.70 |
|  | Microglia | *TNF* | 1.43 | 0.00ⅹ10^0^ | 1.70 | 16.90 |
|  |  | *COMT* | 0.97 | 3.89ⅹ10^-132^ | 2.95 | 35.34 |
|  | Neurons | *GRIN2B* | 3.16 | 0.00ⅹ10^0^ | 10.00 | 82.23 |
|  |  | *GRM5* | 1.80 | 0.00ⅹ10^0^ | 3.25 | 62.18 |
|  |  | *TAC1* | 1.69 | 0.00ⅹ10^0^ | 2.41 | 15.94 |
|  |  | *CNR1* | 0.92 | 0.00ⅹ10^0^ | 1.03 | 25.62 |
|  |  | *BDNF* | 0.64 | 0.00ⅹ10^0^ | 0.65 | 19.10 |
|  |  | *CACNA1C* | 0.58 | 0.00ⅹ10^0^ | 0.57 | 20.20 |

**Table S2.** Significant differentailly expressed genes comparing to other cell types in PNS datasets

| Data | Cluster | Gene | Log2  Fold change | P-value | Average  Expression | Expression  Percentage (%) |
| --- | --- | --- | --- | --- | --- | --- |
| GSE101984 | cLTMR | *COMT* | 0.29 | 6.78ⅹ10^-5^ | 1.41 | 40.92 |
|  | Schwann | *HTR3A* | 0.91 | 7.18ⅹ10^-3^ | 1.54 | 26.67 |
|  | PEP | *TAC1* | 2.51 | 0.00ⅹ10^0^ | 77.80 | 98.46 |
|  |  | *ADCYAP1* | 2.25 | 0.00ⅹ10^0^ | 8.63 | 83.47 |
|  |  | *BDNF* | 0.97 | 1.45ⅹ10^-46^ | 3.53 | 53.73 |
|  |  | *HTR1B* | 0.43 | 3.50ⅹ10^-22^ | 0.60 | 18.07 |
|  |  | *NOS1* | 0.36 | 1.63ⅹ10^-18^ | 0.40 | 11.59 |
|  | NF | *HTR1D* | 0.39 | 6.95ⅹ10^-69^ | 1.18 | 54.76 |
|  |  | *HTR3A* | 0.73 | 4.96ⅹ10^-31^ | 0.24 | 16.67 |
| Mouse  Brain Atlas | cLTMR | *TAC1* | 2.64 | 4.94ⅹ10^-69^ | 29.00 | 93.84 |
|  |  | *ADCYAP1* | 0.27 | 5.66ⅹ10^-6^ | 1.24 | 36.99 |
|  |  | *BDNF* | 0.60 | 7.45ⅹ10^-6^ | 3.00 | 64.38 |
|  | Satellite glia | *SLC1A3* | 0.59 | 7.47ⅹ10^-36^ | 0.55 | 16.67 |
|  | Schwann | *COMT* | 0.99 | 7.19ⅹ10^-07^ | 2.77 | 68.09 |
|  | NP | *MAOA* | 0.27 | 7.37ⅹ10^-57^ | 0.56 | 49.38 |
|  | PEP | *ADCYAP1* | 2.26 | 2.77ⅹ10^-301^ | 4.36 | 81.62 |
|  |  | *TAC1* | 2.28 | 9.80ⅹ10^-130^ | 17.30 | 80.19 |
|  |  | *HTR3A* | 1.04 | 7.86ⅹ10^-84^ | 1.13 | 28.88 |
|  |  | *BDNF* | 1.24 | 7.61ⅹ10^-83^ | 4.16 | 84.73 |
|  |  | *CNR1* | 0.52 | 1.14ⅹ10^-73^ | 0.49 | 27.92 |
|  |  | *HTR1B* | 0.36 | 1.44ⅹ10^-50^ | 0.30 | 17.18 |
|  |  | *CACNA1C* | 0.61 | 8.94ⅹ10^-39^ | 0.98 | 50.60 |
|  |  | *NOS1* | 0.68 | 2.17ⅹ10^-23^ | 0.71 | 21.00 |
|  | NF | *HTR1D* | 0.99 | 5.61ⅹ10^-72^ | 1.18 | 54.76 |
| Mouse GSE197289 | cLTMR | *MAOA* | 2.31 | 0.00ⅹ10^0^ | 266.00 | 78.56 |
|  |  | *HTR1D* | 1.70 | 0.00ⅹ10^0^ | 43.60 | 34.70 |
|  |  | *TAC1* | 1.33 | 0.00ⅹ10^0^ | 463.00 | 80.78 |
|  | Satellite glia | *SLC1A2* | 4.36 | 0.00ⅹ10^0^ | 133.00 | 10.42 |
|  |  | *SLC1A3* | 2.84 | 0.00ⅹ10^0^ | 267.00 | 36.90 |
|  |  | *CACNA1C* | 0.87 | 0.00ⅹ10^0^ | 156.00 | 30.31 |
|  | Schwann | *PRL* | 0.84 | 8.93ⅹ10^-104^ | 102.00 | 10.98 |
|  |  | *COMT* | 1.32 | 1.70ⅹ10^-26^ | 164.00 | 24.45 |
|  |  | *MTHFR* | 0.95 | 4.17ⅹ10^-24^ | 56.70 | 9.33 |
|  | NP | *BDNF* | 1.90 | 0.00ⅹ10^0^ | 105.00 | 53.07 |
|  |  | *MAOA* | 1.27 | 0.00ⅹ10^0^ | 132.00 | 63.73 |
|  |  | *CACNA1C* | 1.21 | 0.00ⅹ10^0^ | 240.00 | 71.92 |
|  |  | *CNR1* | 0.48 | 0.00ⅹ10^0^ | 38.40 | 30.82 |
|  | PEP | *ADCYAP1* | 6.28 | 0.00ⅹ10^0^ | 849.00 | 82.14 |
|  |  | *TAC1* | 5.37 | 0.00ⅹ10^0^ | 3330.00 | 94.38 |
|  |  | *HTR1A* | 3.96 | 0.00ⅹ10^0^ | 25.20 | 13.55 |
|  |  | *BDNF* | 3.36 | 0.00ⅹ10^0^ | 248.00 | 50.61 |
|  |  | *CACNA1C* | 2.15 | 0.00ⅹ10^0^ | 434.00 | 64.24 |
|  |  | *CNR1* | 1.83 | 0.00ⅹ10^0^ | 92.90 | 33.45 |
|  |  | *HTR3A* | 1.65 | 4.86ⅹ10^-291^ | 242.00 | 29.27 |
|  |  | *MAOA* | 1.03 | 1.85ⅹ10^-256^ | 114.00 | 41.94 |
|  |  | *OPRM1* | 2.04 | 3.42ⅹ10^-165^ | 29.60 | 10.28 |
|  | NF | *HTR3A* | 4.00 | 0.00ⅹ10^0^ | 364.00 | 34.28 |
|  |  | *HTR1D* | 3.51 | 0.00ⅹ10^0^ | 57.10 | 25.67 |
|  |  | *CNR1* | 2.94 | 0.00ⅹ10^0^ | 101.00 | 28.29 |
|  |  | *BDNF* | 1.92 | 0.00ⅹ10^0^ | 79.80 | 22.05 |
|  |  | *MAOA* | 0.56 | 2.16ⅹ10^-305^ | 78.20 | 27.91 |
| Human GSE197289 | cLTMR | *HTR3A* | 3.67 | 5.70ⅹ10^-180^ | 98.50 | 51.14 |
|  |  | *HTR1D* | 3.24 | 3.72ⅹ10^-56^ | 32.10 | 28.41 |
|  |  | *CACNA1C* | 2.76 | 5.74ⅹ10^-42^ | 394.00 | 68.18 |
|  |  | *MTHFR* | 0.62 | 2.34ⅹ10^-10^ | 50.70 | 38.64 |
|  |  | *ACE* | 0.83 | 6.21ⅹ10^-7^ | 7.88 | 11.36 |
|  |  | *MAOB* | 0.63 | 6.25ⅹ10^-5^ | 60.10 | 31.82 |
|  |  | *MAOA* | 0.44 | 3.32ⅹ10^-4^ | 21.70 | 20.45 |
|  | Satellite glia | *GRIN2B* | 1.78 | 5.09ⅹ10^-174^ | 81.30 | 20.53 |
|  | Schwann | *COMT* | 0.45 | 3.20ⅹ10^-3^ | 77.20 | 16.00 |
|  |  | *MTHFR* | 0.66 | 3.17ⅹ10^-1^ | 47.10 | 10.67 |
|  | NP | *HTR3A* | 5.57 | 0.00ⅹ10^0^ | 266.00 | 67.24 |
|  |  | *NOS1* | 2.48 | 1.10ⅹ10^-71^ | 90.40 | 36.21 |
|  |  | *CACNA1C* | 2.36 | 5.12ⅹ10^-54^ | 296.00 | 57.47 |
|  |  | *BDNF* | 2.36 | 4.80ⅹ10^-51^ | 16.60 | 18.39 |
|  |  | *ACE* | 1.79 | 2.14ⅹ10^-32^ | 15.90 | 16.09 |
|  |  | *MAOB* | 1.10 | 6.46ⅹ10^-22^ | 83.00 | 39.66 |
|  |  | *MAOA* | 0.33 | 3.28ⅹ10^-10^ | 19.90 | 21.84 |
|  |  | *CNR1* | 1.09 | 9.16ⅹ10^-9^ | 17.10 | 13.79 |
|  | PEP | *TAC1* | 5.04 | 0.00ⅹ10^0^ | 1170.00 | 66.57 |
|  |  | *OPRM1* | 4.65 | 0.00ⅹ10^0^ | 306.00 | 71.29 |
|  |  | *ADCYAP1* | 4.39 | 0.00ⅹ10^0^ | 291.00 | 57.62 |
|  |  | *NOS1* | 3.81 | 0.00ⅹ10^0^ | 135.00 | 41.49 |
|  |  | *CACNA1C* | 2.40 | 0.00ⅹ10^0^ | 253.00 | 57.03 |
|  |  | *MAOB* | 2.01 | 0.00ⅹ10^0^ | 135.00 | 54.57 |
|  |  | *BDNF* | 2.92 | 5.35ⅹ10^-205^ | 18.50 | 15.24 |
|  |  | *SLC1A2* | 0.92 | 1.62ⅹ10^-174^ | 13.90 | 18.09 |
|  |  | *ACE* | 1.03 | 7.53ⅹ10^-67^ | 8.64 | 10.32 |
|  |  | *CNR1* | 0.27 | 1.22ⅹ10^-22^ | 9.27 | 10.42 |
|  | NF | *CNR1* | 3.67 | 0.00ⅹ10^0^ | 41.60 | 22.35 |
|  |  | *HTR1D* | 2.98 | 0.00ⅹ10^0^ | 13.90 | 14.40 |
|  |  | *MAOA* | 1.53 | 0.00ⅹ10^0^ | 37.50 | 27.67 |
|  |  | *MAOB* | 1.31 | 0.00ⅹ10^0^ | 81.20 | 42.67 |
|  |  | *ADCYAP1* | 1.35 | 2.83ⅹ10^-234^ | 62.80 | 25.55 |
|  |  | *OPRM1* | 0.74 | 2.48ⅹ10^-83^ | 44.30 | 19.11 |

**Table S3.** Consistent highly expressed genes across all human datasets, mouse datasets, and all human and mouse datasets

| Species | datasets | Cluster | Gene |
| --- | --- | --- | --- |
| All human |  | Neurons | *GRM5* |
|  |  |  | *CNR1* |
|  |  | Astrocytes | *SLC1A2* |
|  |  |  | *SLC1A3* |
| All mouse |  | Neurons | *BDNF* |
|  |  |  | *GRIN2B* |
|  |  |  | *GRM5* |
|  |  |  | *CNR1* |
|  |  |  | *CACNA1C* |
|  |  | Microglia | *COMT* |
|  |  |  | *TNF* |
|  |  | Astrocytes | *SLC1A2* |
|  |  |  | *SLC1A3* |
|  |  | Endothelial | *MAOA* |
|  |  | NF | *HTR1D* |
|  |  | PEP | *BDNF* |
|  |  |  | *ADCYAP1* |
|  |  |  | *TAC1* |
| All human and mouse |  | Neurons | *GRM5* |
|  |  |  | *CNR1* |
|  |  | Astrocytes | *SLC1A2* |
|  |  |  | *SLC1A3* |
|  |  | NF | *HTR1D* |
|  |  | PEP | *BDNF* |
|  |  |  | *ADCYAP1* |
|  |  |  | *TAC1* |
